# ABCC6 deficiency is associated with activation of BMP signaling in liver and kidney

**DOI:** 10.1016/j.fob.2015.03.009

**Published:** 2015-03-23

**Authors:** Ana M. Blazquez-Medela, Pierre J. Guihard, Jiayi Yao, Medet Jumabay, Aldons J. Lusis, Kristina I. Boström, Yucheng Yao

**Affiliations:** a Division of Cardiology, David Geffen School of Medicine at UCLA, Los Angeles, CA 90095-1679, United States; b Molecular Biology Institute, UCLA, United States

**Keywords:** ABCC6, ATP-binding cassette, subfamily C, member 6, BAEC, bovine aortic endothelial cells, BMPs, bone morphogenetic proteins, MGP, matrix Gla protein, PXE, pseudoxanthoma elasticum, TGFβ, transforming growth factor beta, ATP-binding cassette sub-family C member 6 (ABCC6), Bone morphogenetic protein (BMP), BMP signaling, Mouse, Liver, Kidney

## Abstract

Mutations in ABCC6 (ATP-binding cassette, subfamily C, member 6), an orphan transporter expressed in the liver, are the cause of pseudoxanthoma elasticum. Since ABCC6 was reported to affect matrix Gla protein (MGP), an inhibitor of bone morphogenetic proteins (BMPs), we studied BMP signaling and expression in various tissues of mice with and without functional ABCC. Enhanced BMP signaling was found in all examined tissues in the absence of ABCC6. Despite this, the expression of particular BMP proteins varied widely between tissues. Interestingly, the expression of most BMP proteins in the liver moved in the opposite direction to the same BMP proteins in kidneys in response to ABCC6 alterations. Thus, ABCC6 deficiency stimulates BMP signaling by acting on the expression of multiple BMPs.

## Highlights

• ABCC6 deficiency stimulates BMP signaling in multiple organs.
• ABCC6 deficiency causes tissue-specific induction of BMP-related genes.
• Tissue-specific targeting of BMP signaling may be needed in ABCC deficiency.

## 1 Introduction

ABCC6 (ATP-binding cassette, subfamily C, member 6) is an orphan transporter mainly expressed in the liver [1]. Mutations in the human *ABCC6/MRP6* gene is a known cause of pseudoxanthoma elasticum (PXE) [2], a heritable recessive disorder characterized by calcification of elastin fibers in connective tissue, including the heart, vasculature, skin and eyes [3]. In addition to vascular calcification [4], the mice with *Abcc6* mutations also exhibit other vascular abnormalities including lower elasticity and increased myogenic tone [5]. It is believed that the ABCC6 substrate mediates the ectopic calcification via the circulation since ABCC6 is absent or minimally expressed in the calcified tissue resulting from the deficiency. This is consistent with the finding that parabiotic combination of blood circulation between *Abcc6* knockout *(Abcc6*^−/−^) and wild type mice rescued the vascular calcification [6]. Previous work on ABCC6 deficiency suggests that the tissue effects are mediated by multiple signaling pathways such as the transforming growth factor beta (TGFβ) family and bone morphogenetic proteins (BMPs) [7].

BMP signaling is a pro-mineralizing pathway that has been associated with ABCC6 deficiency and could promote osteochondrogenic transitions in susceptible cells. Indeed, Hosen et al. reported an up-regulation of the BMP2-SMADs-RUNX2 as well as the TGFβ2-SMAD2/3 pathways at the mineralization sites in ABCC6-deficent mice [7], and Meng et al. reported that ABCC6 correlated with the BMP2-Wnt signaling pathway [8]. Matrix Gla protein (MGP) acts as an inhibitor of BMP2, BMP4, BMP6 and BMP7 [9–11] (unpublished data for BMP6), and depends on correctly gamma-carboxylated glutamate residues for optimal function [10]. Low levels of gamma-carboxylation in MGP and low serum levels of MGP have been reported in ABCC6 deficiency [12,13], which might lessen the BMP inhibitory function of MGP. It has also been reported that the gamma-carboxylation system in the vasculature is less efficient than in the liver [14], potentially putting the vasculature at higher risk for calcification if gamma-carboxylation is impaired. Our previous studies suggested that BMP signaling is increased in the ABCC6-deficient heart [15] further supporting that BMP signaling is a downstream target of ABCC6.

Because of the widespread tissue effects of ABCC6 deficiency, we examined if ABCC6, mainly expressed in the liver, affected BMP signaling in other organs, focusing on liver, kidneys, and aorta, and if the same BMP-related genes were activated. We found a general activation of BMP signaling in these organs in the absence of ABCC6. However, the induction of BMP and their receptors varied between tissues, making it difficult to attribute the BMP activation to a particular factor. We conclude that although the presence (or absence) of genetic deficiency of ABCC6 has a systemic effect on BMP signaling, but is caused by tissue-specific induction of BMP-related genes. Targeting BMP signaling for treatment purposes may therefore have to be tailored to the respective organs.

## 2 Methods

### 2.1 Ethics statement

Use of animals and all experimental procedures were review and approved by the University of California, Los Angeles (UCLA) Chancellor’s Animal Research Committee (ARC). The investigation conformed to the National Research Council, Guide for the Care and Use of Laboratory Animals, Eighth Edition (Washington, DC: The National Academies Press, 2011).

### 2.2 Animals and cells

C3H/HeJ (C3H) and C57BL/6J (BL6) mice were purchased from the Jackson Laboratory. *Abcc6*-Tg mice on C3H background were generated as described [8] and contain an *Abcc6* BAC transgene derived from C57BL/6J. *Abcc6* knockout (*Abcc6*^−/−^) mice were obtained from Dr. A.A.B. Bergen [4] and backcrossed for 10 generations on a C57BL/6J background. Littermates were used as wild type controls. All mice were fed a standard chow diet (Diet 8604, HarlanTeklad, Laboratory). All mice were used for experiments at 3–4 months of age.

Bovine aortic endothelial cells (BAEC) were cultured as previously described [16], and BMP4 (0–40 ng/ml, R&D Systems) in culture medium with 10% fetal bovine serum (FBS), or serum from C3H or C3H-*Abcc6*^Tg^ mice was added at the time of plating.

### 2.3 RNA analysis

Quantitative (q)PCR was performed as previously described [16,17]. The primers and probes used for qPCR for mouse activin receptor-like kinase (ALK) 1, ALK2, BMP2, BMP4, BMP6, BMP7, heat shock protein (HSP)70, MGP, Noggin, and crossveinless-2 (CV2) were pre-designed and obtained from Applied Biosystems (Foster City, CA) as part of Taqman® Gene Expression Assays.

### 2.4 Immunofluorescence

Tissue sections were fixed in 4% paraformaldehyde and processed as previously described [18]. Immunofluorescence was performed in detail as previously described [19]. We used specific antibodies for pSMAD1/5/8 (Santa Cruz Biotechnology), total SMAD and ALK2 (both from Santa Cruz Biotechnology), ABCC6 (MRP6 antibody S-20, Santa Cruz Biotechnology), albumin (Bethyl Laboratories). The nuclei were stained with 4′,6-diamidino-2-phenylindole (DAPI) (Sigma–Aldrich) [19].

### 2.5 Immunoblotting

Immunoblotting was performed as previously described [20]. Equal amounts of cellular protein or culture medium were used. Results were then analyzed by immunoblotting using specific antibodies to VEGF (200 ng/ml; R&D Systems). Blots were incubated with specific antibodies to pSMAD1/5/8, pSMAD2/3 (both 400 ng/ml; Cell Signaling Technology), total SMAD, BMP-4, ALK1, ALK2, (all 400 ng/ml; Santa Cruz Biotechnology), BMP-2, ALK3 and ALK6 (all 200 ng/ml; Santa Cruz Biotechnology), BMP-6, BMP-7, BMP receptor type II (BMPRII), CV2, and Noggin (all 200 ng/ml; R&D Systems), HSP70 (100 ng/ml; Stressgen). β-Actin (1:5000 dilution; Sigma–Aldrich) was used as loading control.

### 2.6 Statistical analysis

Data was analyzed for statistical significance by ANOVA with post hoc Tukey’s analysis. The analyses were performed using GraphPad Instat®, version 3.0 (GraphPad Software). Data represent mean ± SD. *P*-values less than 0.05 were considered significant, and experiments were repeated a minimum of three times.

## 3 Results

### 3.1 ABCC6 deficiency is associated with BMP activation in multiple organs

Previous observations suggested that BMP signaling increased in ABCC-deficient hearts [15]. Therefore, we hypothesized that deficiency of ABCC, which is expressed mainly in the liver, had the ability to affect BMP signaling also in other organs.

**Fig. 1:**
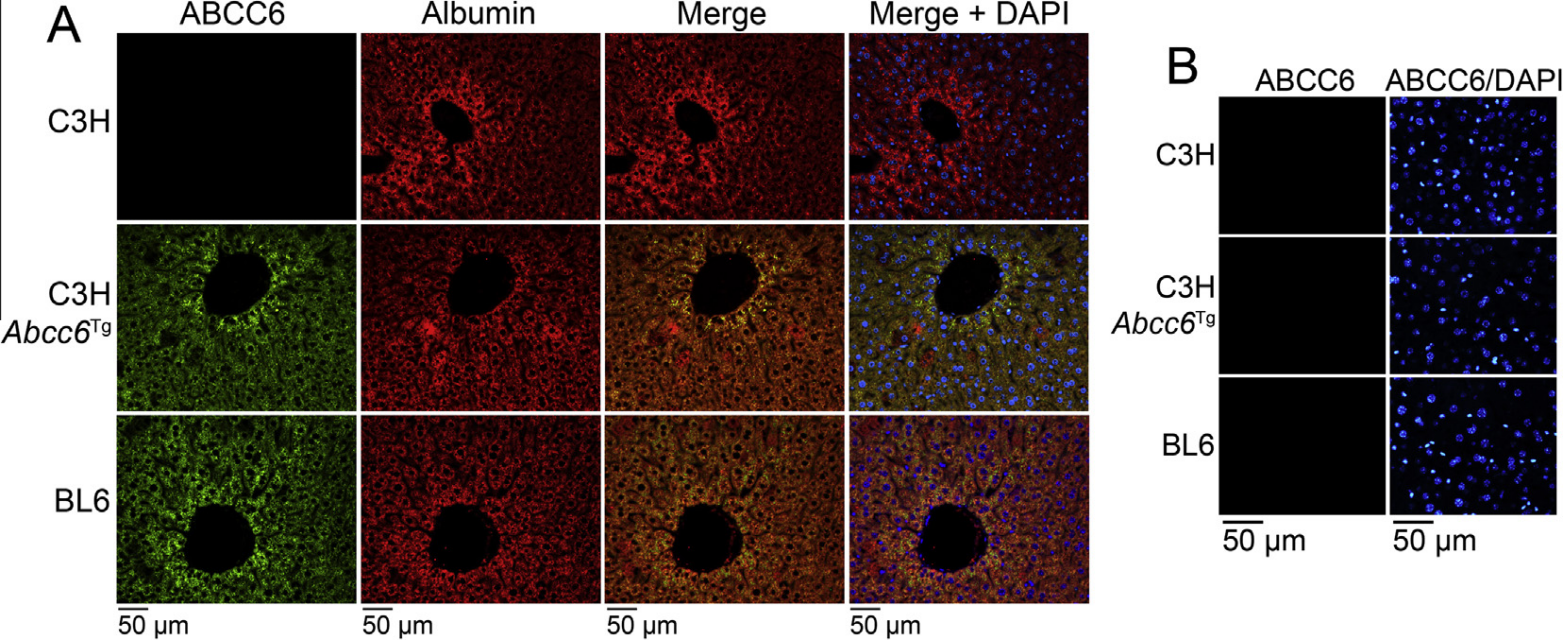
ABCC6 expression in C3H mice without and with an *Abcc6* transgene. (A) Expression of ABCC6 was not detected in the liver of C3H mice (upper panels), but was detected (green staining) in the liver of C3H-*Abcc6*^Tg^ mice (middle panels) and BL6 mice (lower panels). Albumin (red staining) is shown for comparison. DAPI (blue) was used to visualize the cell nuclei. (B) Expression of ABCC6 was not detected in the kidney in C3H, C3H-*Abcc6*^Tg^, or BL6 mice. Tissues from 2 to 3 sets of mice were stained with similar results.

**Fig. 2:**
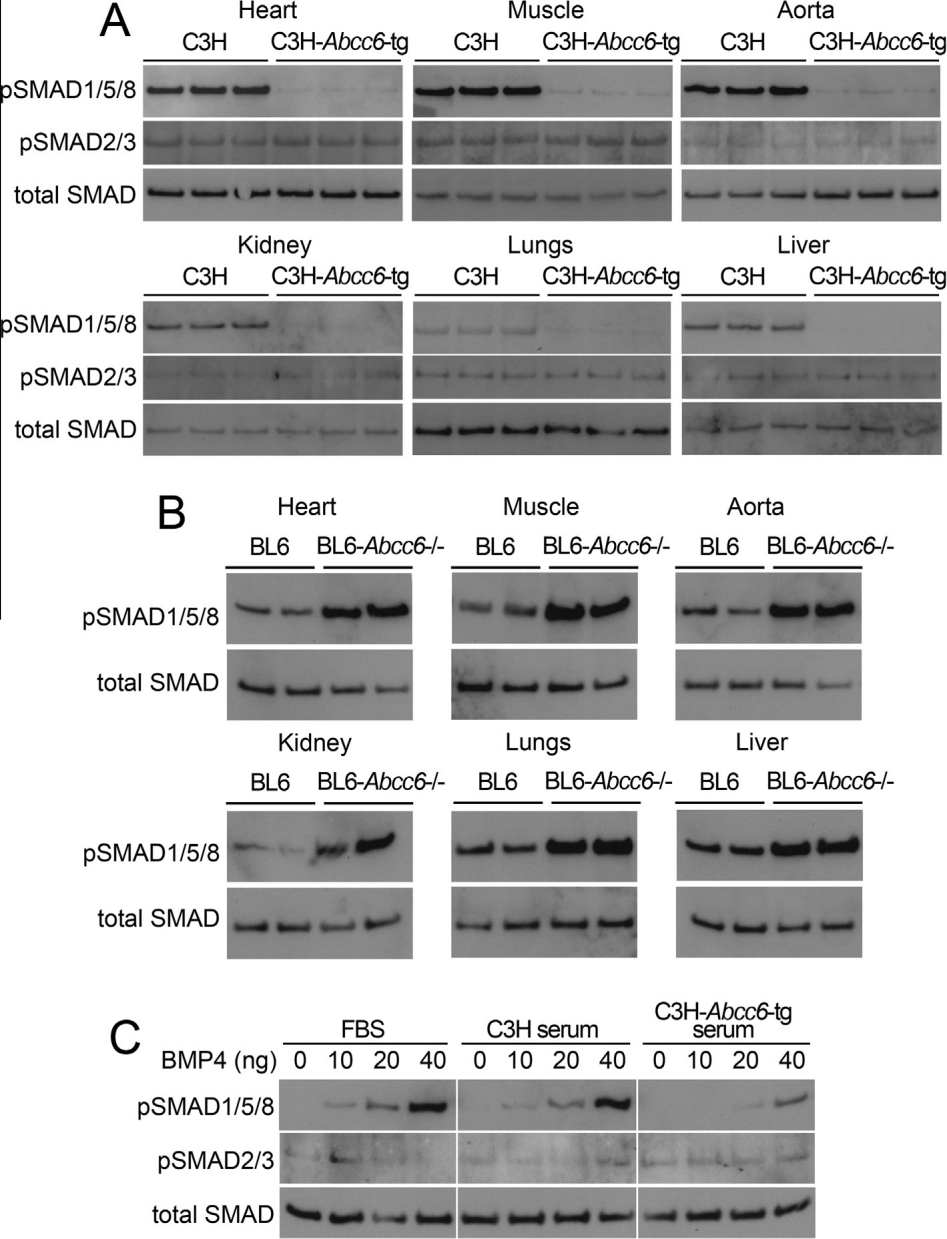
ABCC6 deficiency in C3H and BL6-*Abcc6*^−/−^ mice is associated with BMP activation in multiple organs. (A and B) Phosphorylated (p)SMAD1/5/8 and pSMAD2/3 in heart, muscle, aorta, kidneys, lungs, and liver from (A) C3H and C3H-*Abcc6*^Tg^ mice and (B) BL6 and BL6-Abcc6^−/−^ mice, as determined by immunoblotting and compared to total SMAD (*n* = 3 for each mouse strain). (C) PSMAD1/5/8 and pSMAD2/3 in bovine aortic endothelial cells treated with increasing concentrations of BMP4 (0–40 ng/ml) together with 10% FBS, serum from C3H or serum from C3H-*Abcc6*^Tg^ mice for 24 h, as determined by immunoblotting and compared to total SMAD (*n* = 3 for each mouse strain).

To study BMP signaling, we used C3H wild type mice, which lack functional ABCC6 due to a natural mutation [21], and *Abcc6* transgenic mice on the same background (C3H-*Abcc6*^Tg^ mice). The expression of ABCC6 in liver was about 0.03-fold and 3-fold in C3H and C3H-*Abcc6*^Tg^ mice, respectively, compared to BL6 mice, which express functional ABCC6 as previously reported [8]. We were able to detect ABCC6 by immunofluorescence in the liver of the C3H-*Abcc6*^Tg^ and BL6 mice, but not in the C3H mice (Fig. 1A). Albumin staining is shown for comparison. We were unable to detect ABCC6 expression in other organs by immunofluorescence including kidneys (Fig. 1B), aorta, heart, muscle and lungs (data not shown). We then compared BMP activity in these organs, which revealed that liver, kidneys, heart, aorta, lungs, and muscle all had increased levels of activated phosphorylated (p)SMAD1/5/8 as determined by immunoblotting (Fig. 2A). No changes were detected in the levels of pSMAD2/3, which mediates TGFβ-signaling, or the levels of total SMAD proteins. To confirm these results, we used BL6 wild type mice and BL6 mice with Abcc6 gene deletion (Abcc6^−/−^ mice). As expected, the BL6 mice had less pSMAD1/5/8 in the tested organs when compared to BL6-*Abcc6*^−/−^ mice (Fig. 2B). In all subsequent experiments, we included the BL6 to ensure consistency with the C3H-*Abcc6*^Tg^ mice.

We also compared the ability of serum from C3H and C3H-*Abcc6^Tg^* mice to enhance BMP4 signaling in BAECs. The BAECs were treated for 20–24 h with BMP4 (0–40 ng/ml) in culture medium containing 10% FBS, or serum from C3H or C3H-*Abcc6*^Tg^ mice. The levels of pSMAD1/5/8 were compared by immunoblotting. The results showed that the serum from the C3H-*Abcc6^Tg^* mice caused less activation of SMAD1/5/8 in response to BMP4 than did C3H serum or FBS (Fig. 2C). Together, the results suggest that functional ABCC6 regulates BMP signaling in the organs that were tested, even though ABCC6 expression was only detected in the liver.

### 3.2 Altered expression of ALK2 in presence of the Abcc6 transgene

**Fig. 3:**
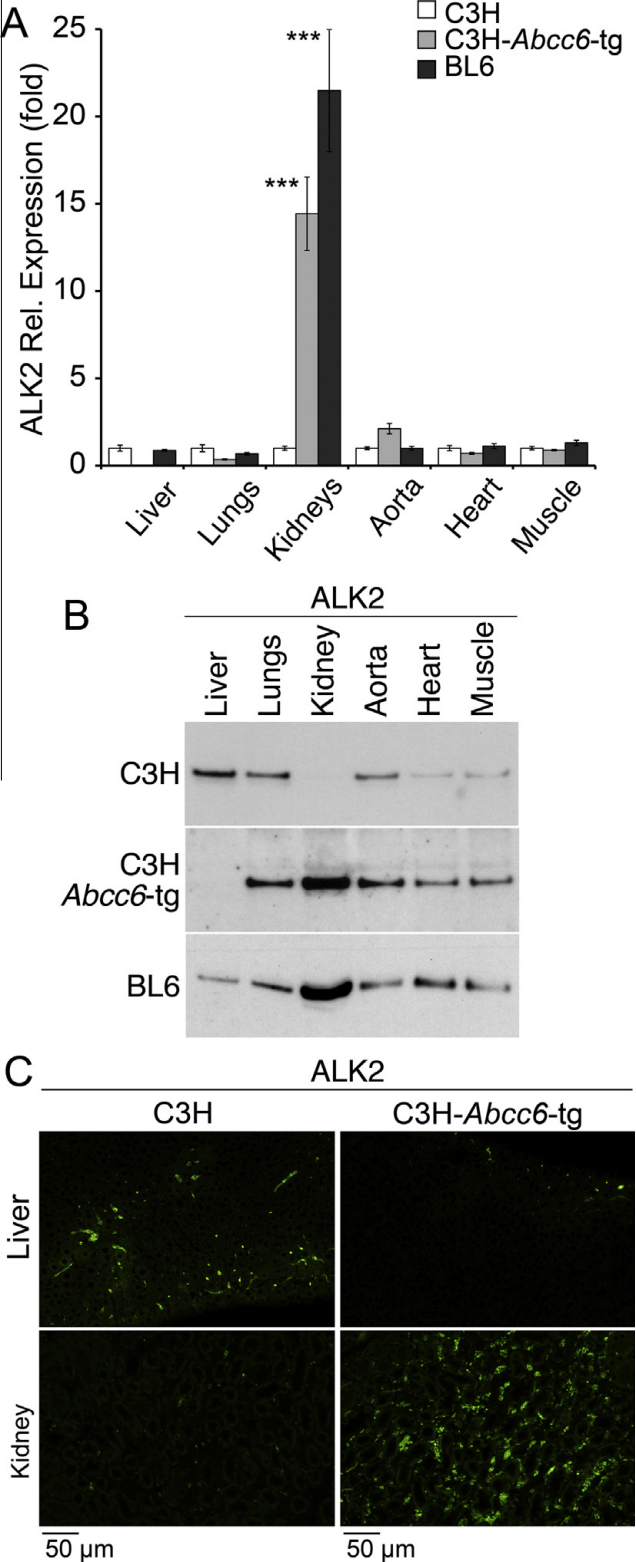
Altered expression of ALK2 in presence of the *Abcc6* transgene in C3H mice. (A and B) Expression of ALK2 in liver, lungs, kidneys, aorta, heart and muscle in C3H mice (without functional ABCC6) and in C3H-*Abcc6*^Tg^ and BL6 mice (both with functional ABCC6), as determined by qPCR (expression is shown relative to that of C3H, which is set at 1) (A) and immunoblotting (B) (*n* = 3 for each mouse strain for qPCR and immunoblotting, respectively). Asterisks indicate statistically significant differences compared to C3H. ^∗∗∗^<0.001, Tukey’s test. (C) Expression of ALK2 in liver and kidneys (green), as determined by immunofluorescence. Tissues from 2 to 3 sets of mice were stained with similar results.

To further examine the BMP activation, we first assessed the expression of ALK2, a type I BMP receptor, in the heart, aorta, muscle, kidneys, lungs and liver of C3H, C3H-*Abcc6^Tg^*, and wild type BL6 mice. ALK2 is widely expressed and can regulate expression of MGP through the ALK1 receptor [22]. The results revealed that the ALK2 expression was particularly enhanced in the kidneys of the C3H-*Abcc6*^Tg^ mice and the BL6 mice, both with functional ABCC6. However, the liver showed the opposite pattern, with significant decreases in ALK2 in the presence of the *Abcc6* transgene, as determined by qPCR and immunoblotting (Fig. 3A and B). The difference in ALK2 expression was also confirmed by immunofluorescence in liver and kidneys (Fig. 3C).

### 3.3 Differential effects on expression of BMP components in liver and kidneys

**Fig. 4:**
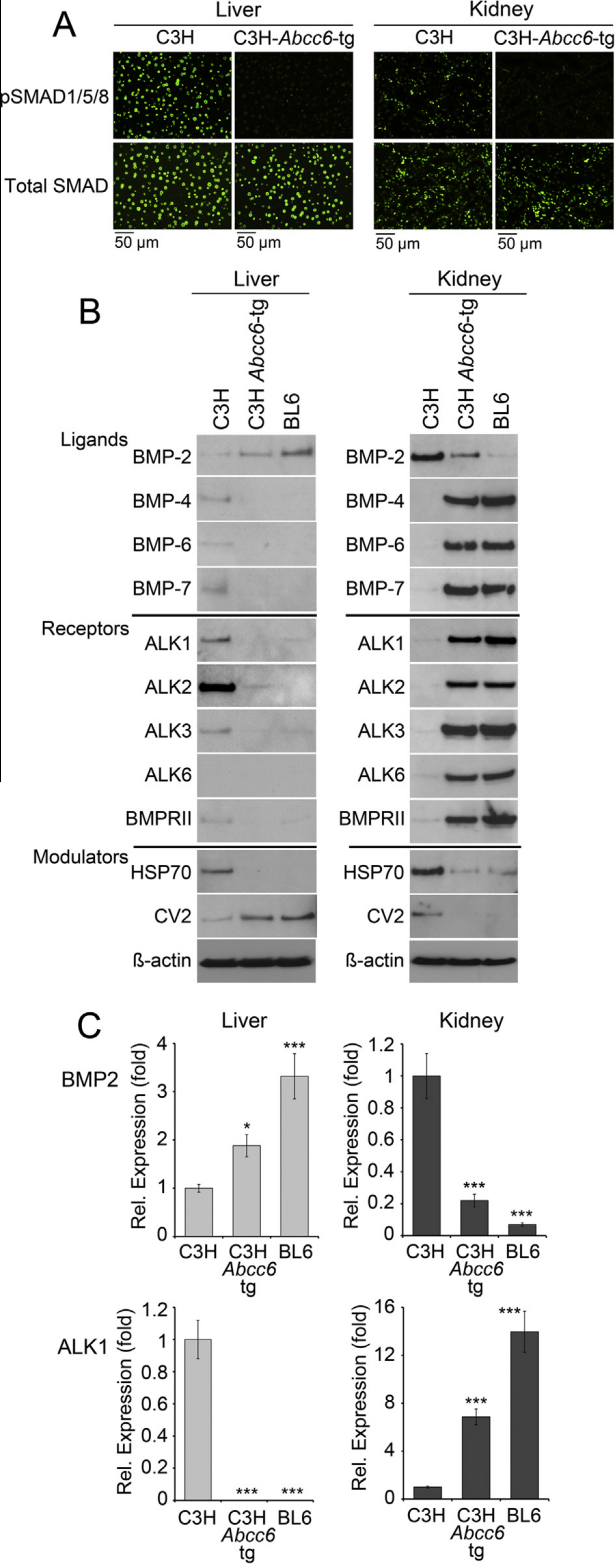
Differential effects on expression of BMP components in liver and kidneys by functional ABCC6. (A) BMP activity in liver and kidney from C3H, C3H-*Abcc6*^Tg^, and BL6 mice, as determined by immunofluorescence for pSMAD1/5/8 (upper panels), and compared to total SMAD (lower panels). Tissues from 2 to 3 sets of mice were stained with similar results. (B) Expression of BMPs, BMP receptors and modulators in liver and kidney from C3H, C3H-*Abcc6*^Tg^, and BL6 mice, as determined by immunoblotting. β-Actin was used as loading control (*n* = 3 for each mouse strain). (C) Expression of BMP2 (top) and ALK1 (bottom) in liver and kidney, from C3H and C3H-*Abcc6*^Tg^ mice, as determined by qPCR (expression is shown relative to that of C3H, which is set at 1) (*n* = 3 for each mouse strain). Asterisks indicate statistically significant differences compared to C3H. ^∗^<0.0, ^∗∗∗^<0.001, Tukey’s test.

Because of the drastic changes in ALK2 expression in kidneys and liver, we focused further experiments on these two organs. We confirmed that pSMAD1/5/8 levels were decreased in both of the organs from the C3H-*Abcc6^Tg^* mice by immunofluorescence (Fig. 4A). We then investigated the expression of the BMPs (BMP2, 3, 6, and 7), which are inhibited by MGP, and the BMP receptors (ALK1, 2, 3, and 6, and BMPRII). We compared expression in the C3H mice with that of the C3H-*Abcc6*^Tg^ mice and BL6 mice using immunoblotting. The results showed different patterns of expression in kidneys and liver of the BMPs and BMP receptors. All of these proteins, except BMP2, increased in the kidneys but decreased or remained about the same in the liver (Fig. 4B). BMP2, on the other hand, decreased in the kidneys and increased in the liver (Fig. 4B). The expression was similar when examined by qPCR, as shown for ALK1 and BMP2 (Fig. 4C), supporting the different patterns of expression of BMPs and their receptors in kidneys versus liver.

We also studied select modulators of BMP activity, including CV2, a BMP inhibitor that may act in tandem with MGP [23], and HSP70, an inflammatory protein that enhances BMP activity by binding to MGP [24]. Similar to BMP2, CV2 decreased in the kidneys and increased in the liver when ABCC6 was functional, whereas HSP70 decreased in both organs (Fig. 4). Despite these changes, the overall pSMAD1/5/8 levels increased similarly in both organs in the absence of functional ABCC6 as described above.

### 3.4 Effect on expression of BMP components in aorta

**Fig. 5:**
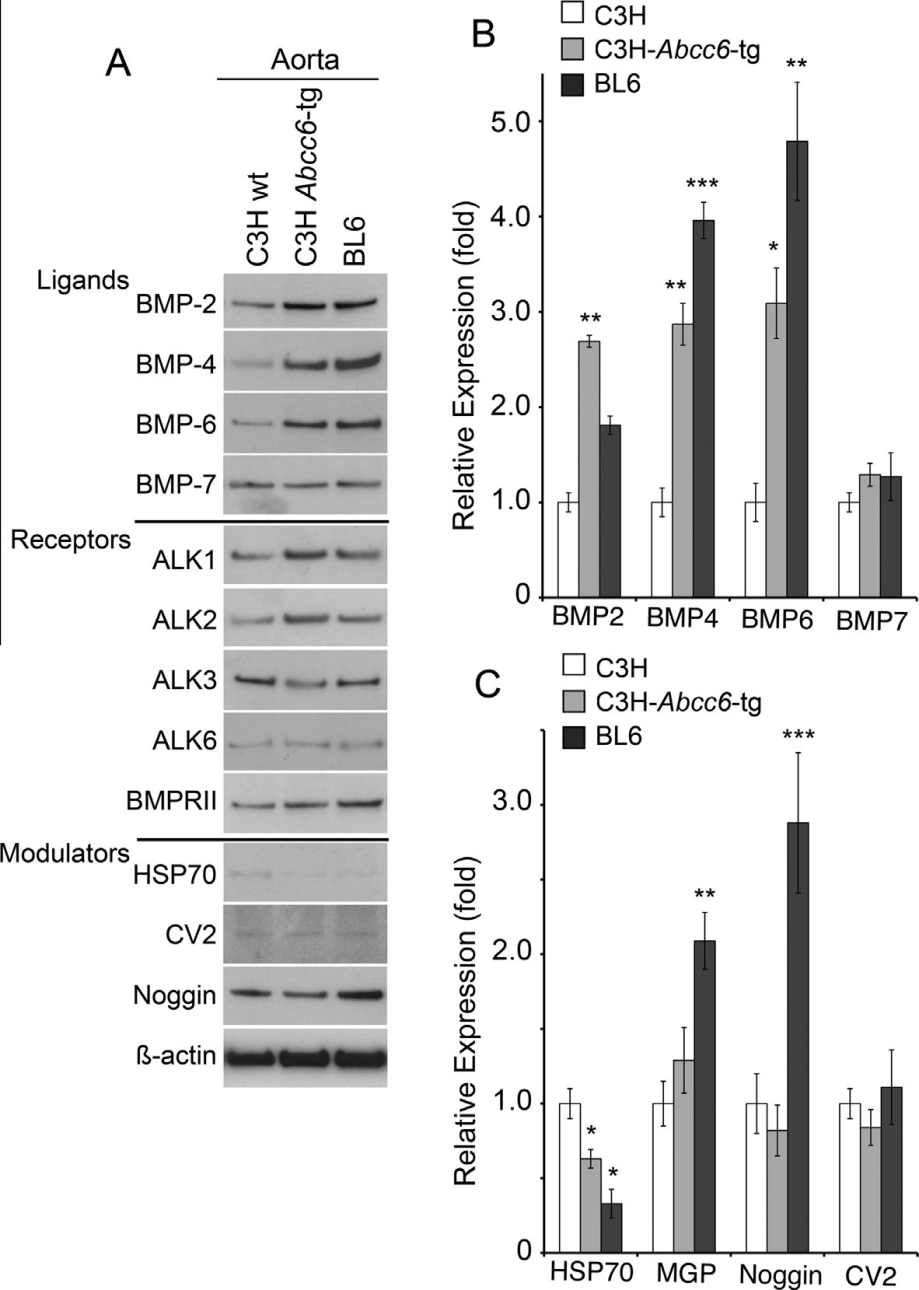
Effect on expression of BMP components in the aorta by functional ABCC6. (A) Expression of BMPs, BMP receptors, and BMP modulators in aortas from C3H, C3H-*Abcc6*^Tg^, and BL6 mice, as determined by immunoblotting. β-Actin was used as loading control. (B and C) Expression of BMPs (B) and BMP modulators (C) in aortas from C3H, C3H-*Abcc6*^Tg^, and BL6 mice, as determined by qPCR (expression is shown relative to that of C3H, which is set at 1) (*n* = 3 for each mouse strain for qPCR and immunoblotting, respectively). Asterisks indicate statistically significant differences compared to C3H. ^∗^<0.0, ^∗∗^<0.01, ^∗∗∗^<0.001, Tukey’s test.

Since lack of ABCC6 has been implicated in vascular calcification, we also examined expression of BMPs, BMP receptors and modulators in the aorta. Again, we compared expression in the C3H mice with that of C3H-*Abcc6*^Tg^ mice and BL6 mice using immunoblotting. The results were less dramatic than in the liver and kidneys, but showed mild increases in BMP-2, -4, and -6 (but not BMP-7) associated with the enhanced ABCC6 levels (Fig. 5A, B), similar to the kidney pattern. Interestingly, it is the opposite of what would be expected considering that vascular calcification is associated with ABCC6-deficient vessels. Therefore, we also looked at the expression of HSP70, MGP, Noggin and CV2 using qPCR. The results showed a mild decrease in HSP70, and mild increases in MGP and Noggin (Fig. 5B and C), which might be enough to limit the BMP activity and protect against calcification, although further studies would be needed to prove this. We were unable to obtain immunoblots for MGP due to unavailability of appropriate antibodies. No significant change was seen in CV2 expression (Fig. 5A and B).

## 4 Discussion

We examined the effect of ABCC6 deficiency on BMP activation and BMP expression in several organs. Together, our results suggest that functional Abcc6, detectable only in the liver in this study, limits BMP activation in all organs studied based on the levels of pSMAD1/5/8. However, the respective expression profiles of BMPs, and BMP receptors and modulators differ between organs, with mostly opposite effects in the liver versus the kidneys. Thus, it is an example of a widespread, systemic effect on one pathway, BMP signaling, which may cause different pathologies depending on the tissue. The ABCC6 effects are likely to be affected by the genetic context in various mouse strains, as already shown by the identification of ABCC6 as the *Dyscalc1* locus [8].

It is not clear why we did not detect ABCC6 by immunofluorescence in the liver and the heart of the C3H mice, even though Aherrahrou et al. [21] detected ABCC6 in the liver by immunoblotting, and Meng et al. [8] detected ABCC6 by qPCR. It is possible that low levels of ABCC6 protein was not detected by the ABCC6 antibodies use in this study.

Alterations in BMP signaling are likely to contribute to the effects of ABCC6 deficiency in cardiovascular system since BMPs have been associated with hypertension, non-laminar flow, endothelial inflammation, angiogenesis, and calcification in the vascular wall as well as the myocardium [15,25–29]. It may also be instrumental in causing pathological changes in skin and eyes, based on previous report demonstrating the importance of BMP signaling in these organs [30,31]. An upregulation of the BMP2-SMADs-Runx2 signaling has been shown to co-localize with mineralization sites in vibrissae and eyes in *Abcc6*^−/−^ mice and in human PXE dermis [7]. There is also an upregulation of BMP2, Runx2 and SMADs in PXE fibroblasts compared to healthy controls [7]. Overall, our findings of enhanced BMP activity in absence of ABCC6 are consistent with previously reported findings.

Our studies are also consistent with previous reports suggesting that ABCC6 in the liver affects other organs at a distance, possibly through a circulating mediator(s). However, the identity and how this putative mediator relates to BMP signaling requires additional research, and is outside the scope of this study. The difficulty of finding this putative mediator may be substantial considering there is still an ongoing discussion of where ABCC6 is located in the cell [32,33] in the mitochondria-associated membranes or the plasma membrane. However, several candidate factors have been identified by other authors, in particular pyrophosphate, a potent inhibitor of vascular calcification that recently was shown been to be decreased in *Abcc6*^−/−^ mice [34,35]. Other investigators have shown that BMP2 can regulate phosphate uptake [36], and that phosphate levels can regulate BMP2 expression [37]. However, it is not clear how pyrophosphate may mediate other ABCC6 effects besides calcification.

Thus, our results suggest that although ABCC6 deficiency activates BMP signaling in multiple organs, the pattern of expression for each BMP component varies. It further suggests that BMP activation is coordinated between different organs, possibly through circulating mediators, and that the organ-specific context determines the effect of ABCC6. The systemic activation of BMP may in part help explain the different features of pseudoxanthoma elasticum, caused by mutations in ABCC6. The differences in gene expression, however, will have to be considered in targeting the BMP signaling for potential treatments. The exact nature of the link between ABCC6 function and BMP signaling is not clear and will require more research. Further studies will also be required to identify transcription factors that might coordinate the effect of ABCC6 on gene expression.

## Author contribution statement

K.I. B, Y. Y conception and design; A.J. L contributed reagents; A. B.-M Collection and assembly of data; A. B.-M , P. G, J, Y, M. J, K. B, Y. Y data analysis and interpretation; A. B.-M., K.I. B, Y. Y manuscript writing; A.J. L, K.I. B, Y. Y financial support.
